# Effects of cholesterol on the size distribution and bending modulus of lipid vesicles

**DOI:** 10.1371/journal.pone.0263119

**Published:** 2022-01-28

**Authors:** Mohammad Abu Sayem Karal, Nadia Akter Mokta, Victor Levadny, Marina Belaya, Marzuk Ahmed, Md. Kabir Ahamed, Shareef Ahammed

**Affiliations:** 1 Department of Physics, Bangladesh University of Engineering and Technology, Dhaka, Bangladesh; 2 Theoretical Problem Center of Physico-Chemical Pharmacology, Russian Academy of Sciences, Moscow, Russia; 3 Department of Mathematics of Russian State University for the Humanities, Moscow, Russia; University of Houston, UNITED STATES

## Abstract

The influence of cholesterol fraction in the membranes of giant unilamellar vesicles (GUVs) on their size distributions and bending moduli has been investigated. The membranes of GUVs were synthesized by a mixture of two elements: electrically neutral lipid 1, 2-dioleoyl-*sn*-glycero-3-phosphocholine (DOPC) and cholesterol and also a mixture of three elements: electrically charged lipid 1,2-dioleoyl-*sn*-glycero-3-phospho-(1′-*rac*-glycerol) (DOPG), DOPC and cholesterol. The size distributions of GUVs have been presented by a set of histograms. The classical lognormal distribution is well fitted to the histograms, from where the average size of vesicle is obtained. The increase of cholesterol content in the membranes of GUVs increases the average size of vesicles in the population. Using the framework of Helmholtz free energy of the system, the theory developed by us is extended to explain the experimental results. The theory determines the influence of cholesterol on the bending modulus of membranes from the fitting of the proper histograms. The increase of cholesterol in GUVs increases both the average size of vesicles in population and the bending modulus of membranes.

## 1 Introduction

Lipid molecules dispersed in buffer solution exhibit a self-assembled system that transforms into the aggregates of various sizes and shapes [1–3]. Vesicles are closed, spherical structures formed by a double lipid layer ranging from nano- to micrometer in diameter. The vesicles are in the center of a huge number of researches because such vesicles are the model of real biological cells [4, 5]. There are several methods of forming the unilamellar vesicles which produce different sizes of vesicles. Among the different unilamellar vesicles, giant unilamellar vesicles (GUVs) of diameters 10 μm or more have attracted special interest due to the visualization of their size and shape using optical microscopes [6, 7]. Such vesicles can be obtained through the natural swelling method [7–10]. The size of GUVs gives the opportunity to study the phenomena happening in a single individual vesicle. The GUVs have been used to investigate the elasticity of lipid membranes [8, 11], rupture/pore formation of vesicles using mechanical/electrical tension [12–15], pore formation due to peptides and nanoparticles [16–18] etc. Such vesicles have been substantially investigated in medical researches as a potential system for delivering the drug to specific body organs [19–22].

It is also worth to mention here that for the preparation of GUVs, the natural swelling method (that we have used here) is a well-accepted process for getting the oil-free different sized GUVs [7]. The analysis of the size distribution of vesicles in such population provides important information about the processes of GUVs formation. There were a number of experimental and theoretical studies dealing with this problem which elucidated the basic principles of the spontaneous lipid vesiculation [23–25]. Currently, it is commonly accepted that the equilibrium size distribution of a vesicle population as well as the stability of each vesicle in the population are determined by a competition between total curvature energy of all vesicles and various sources of entropy of the system, such as vesicle translation and bilayer undulation. Additionally, it was shown that the membrane bending modulus was a key factor that determined vesicle size distributions [25].

In our previous research, the influence of electrostatic conditions (salt concentration of the solution and vesicle surface charge density) on the size distribution of self-assembled GUVs was investigated [25]. It was obtained that the decrease of salt concentration as well as the increase of surface charge density of the membranes increase the average size of GUVs in the population. Based on the analysis of histograms, we showed that the variation of bending modulus due to the changing of electrostatic parameters of the system was the main factor causing to change the average size of GUVs in the vesicle’s population. It is well known that cholesterol is an important component of cell membranes and is present in different membrane types, such as mitochondrial and plasmatic membranes [26]. It plays important role in the functioning of real biological system, varying up to 50 mol% of the total lipid content. Particularly, the cholesterol inhibits the pore formation in the membranes and increases the line tension of membranes [27, 28]. Lysenin (a pore forming toxin) induces pore formation in the lipid membranes in presence of cholesterol [29]. Therefore, the question arises how cholesterol influences on lipid vesicle characteristics. In this paper, we will present the results of the investigation of the effects of cholesterol on GUVs size distribution. The cholesterol has some ordering effects on lipids. The effect of cholesterol on the mechanical properties of lipid membranes is controversial and hence it poses open questions about the interaction mechanism between cholesterol and lipids.

**Fig 1:**
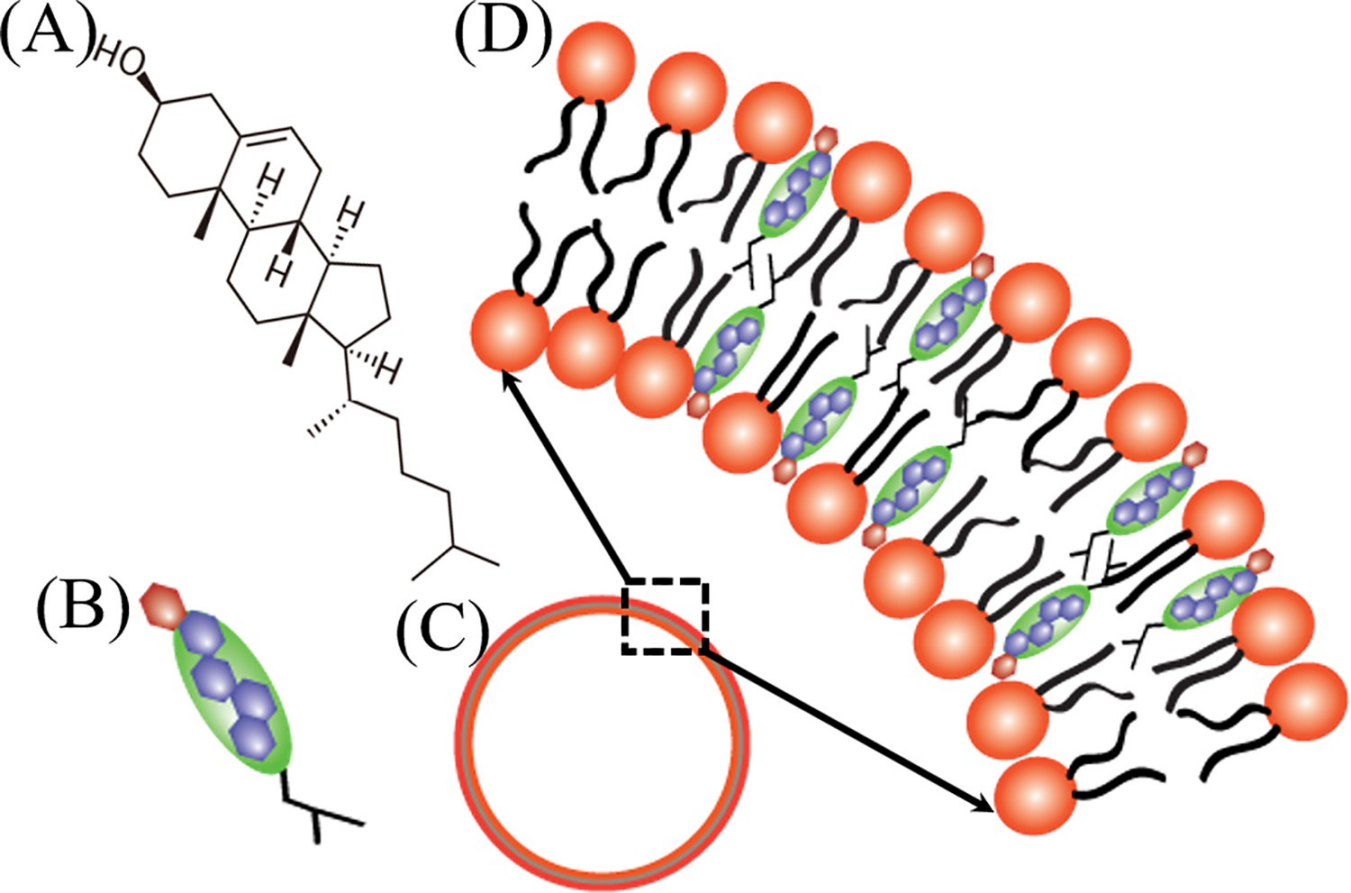
Structure of cholesterol and cholesterol-rich lipid membranes. (A) Structure of cholesterol (B) Illustration of cholesterol (C) Lipid membrane of GUV and (D) Cholesterol-rich lipid membrane.

The bending modulus is the most important parameter determining the mechanical property of lipid membranes. There are many studies on the effects of cholesterol on the mechanical properties of lipid membranes using different experimental techniques, such as micromanipulation, tether pulling, vesicle electrodeformation, nuclear magnetic resonance, X-ray diffraction, etc [30, 31]. But despite bulk studies, presently there is no commonly accepted understanding on how cholesterol influences the bending modulus of bilayer. From several papers, it is known that cholesterol increases the membrane bending rigidity [32–38]. Besides, it was shown a trend of increasing vesicle bending modulus with increasing cholesterol content, up to a 3–4 fold increase at 50 mol% cholesterol [39]. It was demonstrated that the effect of cholesterol on bilayer bending modulus is not universal, but rather it is lipid-specific [40, 41]. In particular, it was obtained that the bending modulus of DOPC membranes does not change significantly with the addition of cholesterol, but the sphingomyelin membranes become more flexible [42]. Values of the bending modulus were reviewed, and possible causes for the considerable differences were discussed [43]. The structure of cholesterol and cholesterol-rich lipid membranes are illustrated in Fig 1.

Very recently, the effect of cholesterol on the bending modulus of DOPC membranes has been reported [44]. DOPC is basically a cis-unsaturated lipid which is a synthetic representative of the class of phosphatidylcholines. Three comprehensive examinations such as neutron spin-echo (NSE) spectroscopy, nuclear magnetic resonance (NMR) spectroscopy and real-space fluctuation (RSF) analysis of atomistic molecular dynamics (MD) simulations have been performed for measuring the bending rigidity. These three techniques separately indicated the increase of bending rigidity with the increase of cholesterol content in the membranes of DOPC vesicles. After publishing a recent work [44], the above finding has been a controversial topic [45, 46]. Later, a paper has been proposed to reconcile this controversy [47].

Bassereau’s group [48] and Baumgart’s group [49] studied tubules, Dimova’s group [42] studied the shape fluctuation analysis (SFA) and electro-deformation (ED) in GUVs, and Nagle group [41] studied X-ray diffuse scattering (XDS) from stacks of bilayers for measuring the bending rigidity of DOPC membranes. All these groups obtained unchanged bending modulus of DOPC membranes with cholesterol. They have considered equilibrium analysis where they used the principles of equilibrium statistical mechanics in their theories. In contrast, the data obtained from NSE, NMR and MD simulations [44] followed decay rates which principally involved non-equilibrium quantities. By considering the accessible length and time scales of different measurement techniques, the discrepancy of results obtained by different groups can be resolved.

In our case, the theory was developed by considering the dynamics of vesicle formation from lipid bilayer aggregates [25]. We assumed that the system (population of lipid molecules) passes a set of states with various size distributions of lipid structures before reaching the final equilibrium state containing the final GUV’s size distribution. In this report, the theory is extended for neutral and charged membranes containing various concentrations of cholesterol. The focus of this research is to investigate the size distribution of DOPC-GUVs (i.e., neutral vesicles) and DOPG/DOPC-GUVs (i.e., charged vesicles) at various cholesterol concentrations in their membranes and to estimate the bending modulus using theory from their corresponding size distributions. As far we know, there is no report for obtaining the bending rigidity of membranes containing cholesterol from the size distribution of giant vesicles observed by an optical microscope. From these investigations, it has been possible to obtain comprehensive understanding of the biological function of cholesterol in membranes and its role in health and disease.

In this paper, at first the experimental methods as well as the used biochemical materials are described. Then the experimental results are presented in which the results are analyzed statistically using lognormal distribution [50]. The obtained experimental results are explained based on the physical theory. Finally, the discussion and conclusions are presented.

## 2 Materials and methods

### 2.1 Chemicals and reagents

1,2-dioleoyl-*sn*-glycero-3-phospho-(1′-*rac*-glycerol) (sodium salt) (DOPG), 1, 2-dioleoyl-*sn*-glycero-3-phosphocholine (DOPC) and 1,2-dipalmitoleoyl-*sn*-glycero-3-phosphocholine (POPC) were purchased from Avanti Polar Lipids Inc. (Alabaster, AL). Bovine serum albumin (BSA), 1,4-Piperazinediethanesulfonic acid (PIPES), Ethylene glycol-bis(2-aminoethylether)-*N*,*N*,*N*′,*N*′-tetraacetic acid (EGTA) were purchased from Sigma-Aldrich (Germany). Cholesterol was purchased from WAKO pharmaceuticals (Japan). Chemicals and reagents were used without further purification.

### 2.2 Synthesis, formation and observations of GUVs

There are several different methods for the formation of GUVs [9, 51–53]. Here to prepare the population of lipid GUVs we used the natural swelling method [7–10]. At first, 200 μL of a mixture of 1 mM DOPC or POPC and cholesterol (or a mixture of 1 mM DOPG, DOPC and cholesterol) was taken into a 4.5 mL glass vial which was gently shacked and kept for 40–60 s to get the compositionally homogeneous mixture of the lipids and cholesterol throughout the total suspension. Due to high diffusion of lipid molecules in chloroform, they are distributed very quickly throughout the bulk of the sample. Then this mixture was dried with a gentle flow of N_2_ gas to produce a thin, homogeneous lipid film followed by the vial that was placed in a vacuum desiccator for 12 hours. During this procedure, the lipid bilayer stacks formed in the vial. After this, a 20 μL MilliQ water was added into the vial and pre-hydrated for 8 minutes at 45°C and then the sample was incubated for 3.5 h at 37°C with 1 mL MilliQ containing 0.10 M sucrose (for neutral membrane) and 1 mL buffer (10 mM PIPES, 150 mM NaCl, pH 7.0, 1mM EGTA) containing 0.10 M sucrose (for charged membrane). As a result, the GUVs with different sizes contained sucrose solution in their inside. We have considered early the problem of the GUV’s vesiculation [7, 17, 25] and concluded that this time is enough for forming the thermodynamically equilibrium population of GUVs. After incubation, the GUV suspension was centrifuged at 13000×g (here g is the acceleration due to gravity) for 20 minutes at 20°C using a refrigerated centrifuge (NF 800R, NUVE, Turkey) for removing the multilamellar vesicles (MLVs) and lipid aggregates. We collected the supernatant after centrifugation for experiment. To minimize the film defect, we always prepared GUVs in 2–3 glass vials at the same time for each independent experiment and took the data from equilibrium population of GUVs. In addition, high centrifugation may also minimize the film defected GUVs. To observe the population of GUVs in a phase contrast microscope, an amount of 280 μL 0.10 M glucose containing MilliQ (for neutral membranes) and 0.10 M glucose containing buffer (for charged membranes) was added into the microchamber. Then 20 μL aliquot of GUVs suspension was introduced into the handmade microchamber and it was waited 20−25 minutes for achieving the equilibrium settle down of vesicles at the bottom of the microchamber. The asymmetrical concentration of sugar between the inside and the outside of GUVs was created for the visualization of GUVs. For removing the strong attraction between the glass surface and the GUVs, the microchamber and the glass surface were coated with 0.10% (w/v) BSA dissolved in the same solution. An inverted phase contrast microscope (Olympus IX-73, Japan) with 20× objective at 25 ± 1°C was used to observe the GUVs and the images were recorded using a charge-coupled device camera (Olympus DP22, Japan). Before going to the next section, it is necessary to clarify the notation used. In particular, the DOPG/DOPC/chol (46/39/15)-GUVs means that in the sample there were 46% of DOPG, 39% of DOPC and 15% of cholesterol, where % indicates the mole%.

To prepare the cholesterol (i.e., chol, *C*_h_)-rich neutral membranes, DOPC/chol (100/0, 100/0 indicates molar ratio), DOPC/chol (85/15), DOPC/chol (71/29), DOPC/chol (60/40), POPC/chol (100/0), POPC/chol (85/15), POPC/chol (71/29) and POPC/chol (60/40)-GUVs were prepared in MilliQ water containing 0.10 M sucrose as an internal solution. As for the preparation of GUVs with charged membranes containing cholesterol, it is necessary to make a note. Embedding in a lipid monolayer, cholesterol condenses it and thereby changes the area per lipid molecule of this monolayer. To obtain GUVs with more or less same surface charge density in case of the charged GUVs with the different fractions of cholesterol (that is necessary for comparison of the results of different experiments) it is necessary to follow some specific procedures. It is well reported that with the addition of cholesterol in the lipid membranes its condensation occurs [54–57]. In the absence of cholesterol the cross sectional area of DOPG (*a*_DOPG_) and DOPC (*a*_DOPC_) lipid molecules is about 72.5 Å^2^/molecule [58]. However, in the presence of cholesterol the cross sectional area of these molecules decreases to about 50, 42 and 40 Å^2^/molecule for 15, 29 and 40 mole% cholesterol, respectively [59, 60]. The cross sectional area of cholesterol molecule is about half of that DOPG or DOPC lipid i.e., 33−38 Å^2^/molecule [55]. The surface charge density of cholesterol-free DOPG/DOPC/chol (70/30/0)-GUVs, Ω_PG_ = *eX*/*a*_DOPG_ is − 0.154 C/m^2^, where *X* is the DOPG mole fraction in the membranes and *e* is electronic charge. The surface charge density of cholesterol containing charged GUVs was determined by expression $\Omega_{\mathrm{ch}}=\frac{Z}{S_{\mathrm{GUV}}}=\frac{eXN_{1}}{a_{\mathrm{DOPG}}(1-c_{h})N_{1}+a_{\mathrm{ch}}c_{h}N_{1}}=\frac{eX}{a_{\mathrm{DOPG}}(1-c_{h})+a_{\mathrm{ch}}c_{h}}$, where *Z* = *XN*_1_ is the number of the charges at GUV’s surface, *S*_GUV_ is the square of GUV’s surface, *N*_1_ is the total number of molecules in GUV’s monolayer and *c*_h_ = *C*_h_/100 is the mole fraction of cholesterol in GUVs. Therefore, to obtain the cholesterol containing charged GUVs with more or less same surface charge density (≈ − 0.15 to − 0.16 C/m^2^) for different fractions of cholesterol in the GUVs with different composition (namely DOPG/DOPC/chol (46/39/15), DOPG/DOPC/chol (43/28/29) and DOPG/DOPC/chol (40/20/40)) were prepared in the buffer containing 0.10 M sucrose as an internal solution. The total salt concentration, *C*, in the buffer was 162 mM [25]. The values of *X* in the DOPG/DOPC/chol (70/30/0), DOPG/DOPC/chol (46/39/15), DOPG/DOPC/chol (43/28/29) and DOPG/DOPC/chol (40/20/40)-GUVs were 0.70, 0.46, 0.43 and 0.40, respectively. Hence all these GUVs with 15, 29 and 40 mole% cholesterol have approximately the same surface charge density (≈ − 0.15 to − 0.16 C/m^2^C/m^2^), which is close to the surface charge density in the membranes of DOPG/DOPC/chol (70/30/0)-GUVs.

## 3 Results and observations

### 3.1 Effects of cholesterol on the size distribution of GUVs of neutral membranes

**Fig 2:**
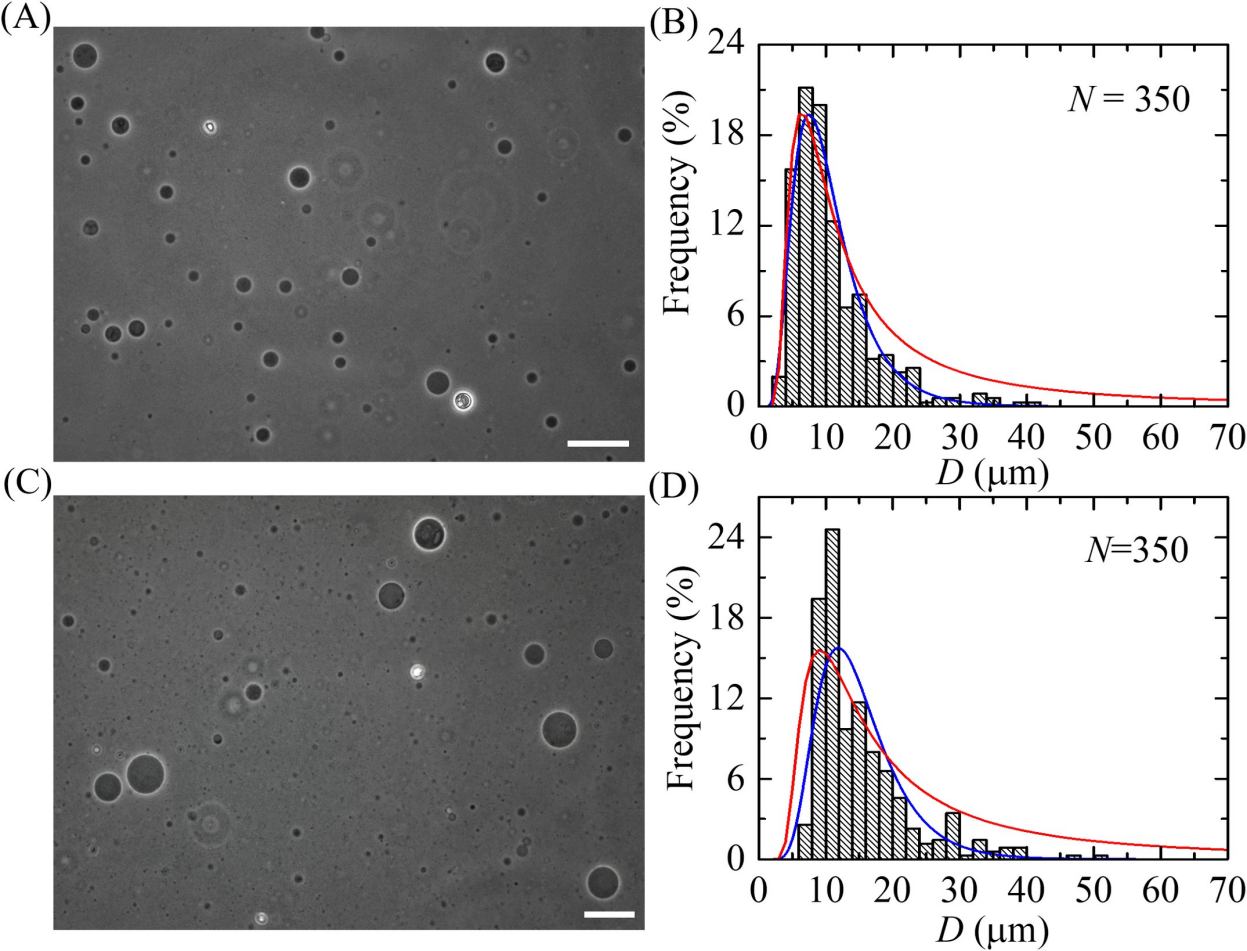
Effects of cholesterol on the size distribution of GUVs containing neutral membranes. (A) and (B) show a phase contrast image and a size distribution histogram of DOPC/chol (100/0)-GUVs, respectively. (C) and (D) show a phase contrast image and a size distribution histogram of DOPC/chol (71/29)-GUVs, respectively. The bar in the images corresponds to 50 μm. *N* is the number of measured GUVs in each independent experiment. The blue lines show the best fitting curves of Eq 1. The parameters in (B) are *μ* = 2.25, *σ* = 0.49, and in (D) are *μ* = 2.63, *σ* = 0.39. The values of the coefficient of determination, *R*^2^, are obtained 0.91 in (B) and 0.95 in (D) from the fitted (blue) curves. The red lines show the best fitting theoretical curves corresponding to the Eq 7. The fitting parameters in (B) are *K*_ben_ = 19.0 *k*_B_*T*, *D*_freq_ = 6.4 μm, *L* = 3535, and in (D) are *K*_ben_ = 28.5 *k*_B_*T*, *D*_freq_ = 9.1 μm and *L* = 4230. The values of *R*^2^ in (B) and (D) are obtained 0.92 and 0.69, respectively from the fitted (red) curves.

To investigate the effects of cholesterol on the size distribution of GUVs in vesicle population, primarily we considered the GUVs with neutral membranes using various molar ratios of *C*_h_. Fig 2 shows the experimental results of DOPC/chol (100/0) and DOPC/chol (71/29)-GUVs. The phase contrast image of DOPC/chol (100/0)-GUVs in the suspension (i.e for *C*_h_ = 0) is shown in Fig 2A. After measuring the diameters, *D*, of *N* = 350 GUVs (i.e., *N* is the number of measured GUVs) from the several phase contrast images of DOPC/chol (100/0)-GUVs, a histogram of the size distribution of GUVs was obtained (Fig 2B). In each experiment, there were more or less 350 GUVs which was chosen arbitrary from the entire ensemble. This amount is quite enough to obtain a representative histogram and to run the statistical analysis. It is seen that the shape of the histogram is asymmetric with positive skewness, indicating a large fraction of small GUVs with 3–10 μm diameters (which is smaller than GUVs of the average diameter) and a small fraction of more than 10 μm diameters GUVs. Note that the similar results have been obtained for others systems (for DOPC-GUVs [25] and for POPC/cholesterol-GUVs [61]. One can assume that such a size distribution of vesicles is a characteristic property of populations of lipid GUVs. To get the mean of the average values of the distribution parameters, we repeated the experiment 12–16 times containing about 350 GUVs in each experiment and got the similar result (i.e., the total number of experiments was *n* = 12–16). Moreover, the conditions in all experiments were same. It was divided all investigated vesicles into a few groups which gave us the opportunity to analyze the obtained results in the framework of statistical analysis of grouped data [62, 63].

Then, we studied the GUVs with cholesterol containing membranes. The results of this experiment (phase contrast image for DOPC/chol (71/29), i.e., *C*_h_ = 29) is shown in Fig 2C. The histogram based on the 350 GUV’s observations (i.e., *N* = 350) from several phase contrast images has been constructed (Fig 2D). It is seen that as in case of DOPC/chol (71/29)-GUVs the shape of histogram is also asymmetric, indicating a large number of more than 11 μm diameters GUVs and a small number of 3–11 μm diameters GUVs. Similar results were also obtained for 12–16 independent experiments. Then the statistical analysis of all obtained data was performed. Thus, the mean of the average size of GUVs was obtained from 4200–5600 GUVs. It is worth to mention that the average size of the distribution of several independent experiments was significantly similar. The standard deviation of the mean of the average size of GUVs was significantly small, which makes us confident to present the data. Similar approach was followed in our several recent papers [7, 25, 64, 65]. Here, we presented only one independent experiment in each condition. The average value *D*_ave_ (i.e., the arithmetic mean over all measured GUVs) from 12–16 independent experiments is shown in Fig 4. By comparing the histograms of Fig 2B and 2D one can conclude that with the increase of *C*_h_ the size distribution of GUVs shifts in the range of larger vesicles, indicating the decrease of histogram asymmetricity. The GUVs size distribution for cholesterol-rich neutral GUVs was also analyzed for two other concentrations namely *C*_h_ = 15 and 40.

To analyze the experimental results quantitatively we use a well-known lognormal distribution [50]:

$$f(D)=\frac{1}{D}\frac{1}{\sigma \sqrt{2\pi }}\exp[-\frac{{\left\{\ln(D)-\mu \right\}}^{2}}{2\sigma^{2}}]$$

$$=\frac{1}{D}\frac{1}{\sigma \sqrt{2\pi }}\exp[-\frac{{\left\{\ln(D/\rho )\right\}}^{2}}{2\sigma^{2}}],$$ (1)

where *f*(*D*) indicates the **probability density function** (frequency of GUVs with diameter *D***)**, the dimension median *ρ* (or dimensionless *μ* = ln *ρ*) and *σ*^**2**^ are the distribution parameters, *μ* is a mean of distribution of (ln*D*). The average value (diameter) of the distribution, *D*_**ave**_, is calculated using Eq 1 as follows [50]:

$$D_{\mathrm{ave}}=\int_{0}^{\infty }Df(D)dD=\exp(\mu +\frac{1}{2}\sigma^{2})=\rho \exp(\frac{\sigma^{2}}{2})$$ (2)

Note that the similar approach based on this distribution was used for the description of the distribution of GUVs suspension at various conditions [7, 25]. The histograms of Fig 2B and 2D are fitted (blue lines) with Eq 1 and the average diameters of the GUVs were obtained using Eq 2. In the first independent experiment, the average sizes (diameters) of GUVs, *D*_ave1_, were obtained 10.7 μm for DOPC/chol (100/0)-GUVs and 15.0 μm for DOPC/chol (71/29)-GUVs. Then, similar experiments were performed and the average sizes of GUVs, *D*_ave2_, were obtained 9.9 μm for DOPC/chol (100/0)-GUVs and 14.9 μm for DOPC/chol (71/29)-GUVs from the second independent experiment. The mean of the average sizes of GUVs, *D*_ave_, was calculated from the results of 12–16 independent experiments (i.e., *n* = 12–16) using more or less *N* = 350 GUVs in each experiment. The values of *D*_ave_ were obtained (10.9 ± 1.0) μm and (14.3 ± 1.4) μm (± indicating the standard deviation) for DOPC/chol (100/0)-GUVs and DOPC/chol (71/29)-GUVs, respectively. Similarly, the values of *D*_ave_ were obtained (11.8 ± 1.1) μm and (15.3 ± 1.4) μm for DOPC/chol (85/15)-GUVs and DOPC/chol (60/40)-GUVs, respectively.

### 3.2 Effects of cholesterol on the size distribution of GUVs of charged membranes

**Fig 3:**
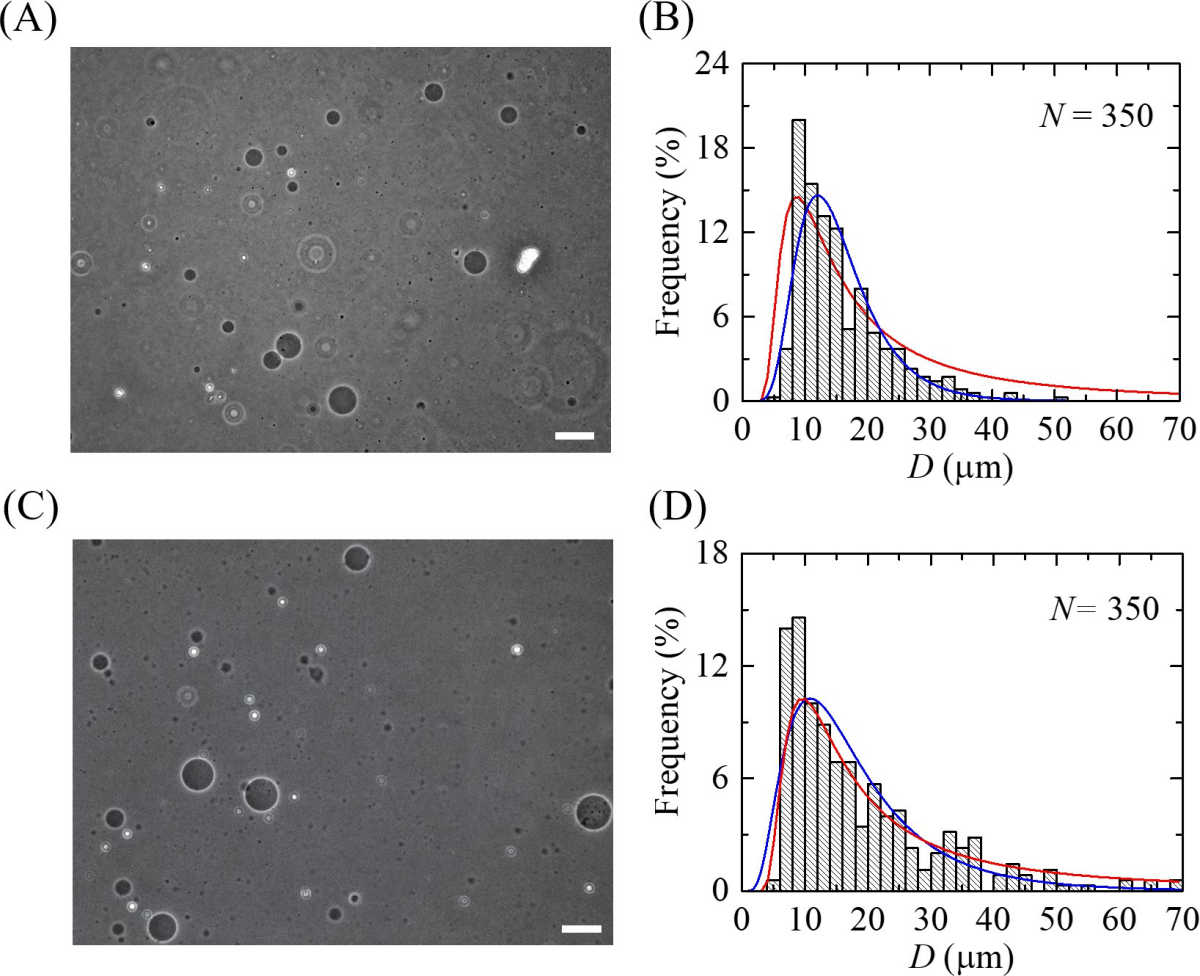
Effects of cholesterol on the size distribution of charged GUVs. (A) and (B) show a phase contrast image and a size distribution histogram of DOPG/DOPC/chol (46/39/15)-GUVs, respectively. (C) and (D) show a phase contrast image and a size distribution histogram for DOPG/DOPC/chol (40/20/40)-GUVs, respectively. The bar in the images corresponds to 50 μm. *N* is the number of measured GUVs in each independent experiment. The blue lines show the best fitting theoretical curves corresponding to Eq 1. The parameters in (B) are *μ* = 2.66, *σ* = 0.42, and in (D) are *μ* = 2.74, *σ* = 0.60. The values of *R*^2^ are obtained 0.88 in (B) and 0.84 in (D) for the fitted (blue) curves. The red lines show the theoretical curves of Eq 7. The parameters in (B) are *K*_ben_ = 26.6 *k*_B_*T*, *D*_freq_ = 8.6 μm, *L* = 3700, and in (D) are *K*_ben_ = 36.8 *k*_B_*T*, *D*_freq_ = 9.5 μm and *L* = 3600. The values of *R*^2^ in (B) and (D) are obtained 0.76 and 0.86, respectively for the fitted (red) curves.

In our previous paper, we have demonstrated that the electrostatic effects influence significantly on the size distribution of GUVs [25]. It is the reason why we also considered here the effect of cholesterol on such distribution in case of GUVs containing charged lipids. The effects of cholesterol in case of charged vesicles membranes on the size distribution of GUVs were investigated upon fixing the surface charge density of membranes (≈ − 0.15 to − 0.16 C/m^2^, see discussion above). We have analyzed the system by varying cholesterol fraction from *C*_h_ = 0 to 40% at *C* = 162 mM. Fig 3 shows the experimental results for DOPG/DOPC/chol (46/39/15) and DOPG/DOPC/chol (40/20/40)-GUVs. A typical experimental result of the phase contrast image of GUVs suspension for DOPG/DOPC/chol (46/39/15)-GUVs is shown in Fig 3A. The corresponding histogram of the size distribution of GUVs using *N* = 350 is shown in Fig 3B. It is seen that in case of DOPG/DOPC/chol (46/39/15)-GUVs the histogram is asymmetrical with the positively skewed distributions, i.e., there are a small number of GUVs with diameters greater than 14 μm and a large number of GUVs with diameters 3–14 μm. Similar results were also obtained in other independent experiments.

A typical phase contrast image in the same buffer for DOPG/DOPC/chol (40/20/40)-GUVs and the corresponding histogram (using *N* = 350) are shown in Fig 3C and 3D, respectively. In this histogram, a large number of GUVs with diameters greater than 15 μm and a small number of GUVs with diameters 3–15 μm are observed. Similar results were obtained from other experiments. Therefore, with the increase of cholesterol the peak of the histogram of the vesicle distribution shifts to the region of the large GUVs. The histograms of Fig 3B and 3D are fitted (blue line) with Eq 1, and the diameters of the GUVs distribution were obtained 15.6 and 18.5 μm using Eq 2 for DOPG/DOPC/chol (46/39/15) and DOPG/DOPC/chol (40/20/40)-GUVs, respectively. The average sizes of GUVs were obtained (14.5 ± 1.3) μm and (16.8 ± 1.3) μm for DOPG/DOPC/chol (46/39/15) and DOPG/DOPC/chol (40/20/40)-GUVs, respectively using 12–16 independent experiments. Similarly, the average sizes of GUVs were obtained (13.9 ± 2.5) μm and (15.7 ± 1.9) μm for DOPG/DOPC/chol (70/30/0) and DOPG/DOPC/chol (43/28/29)-GUVs, respectively.

**Fig 4:**
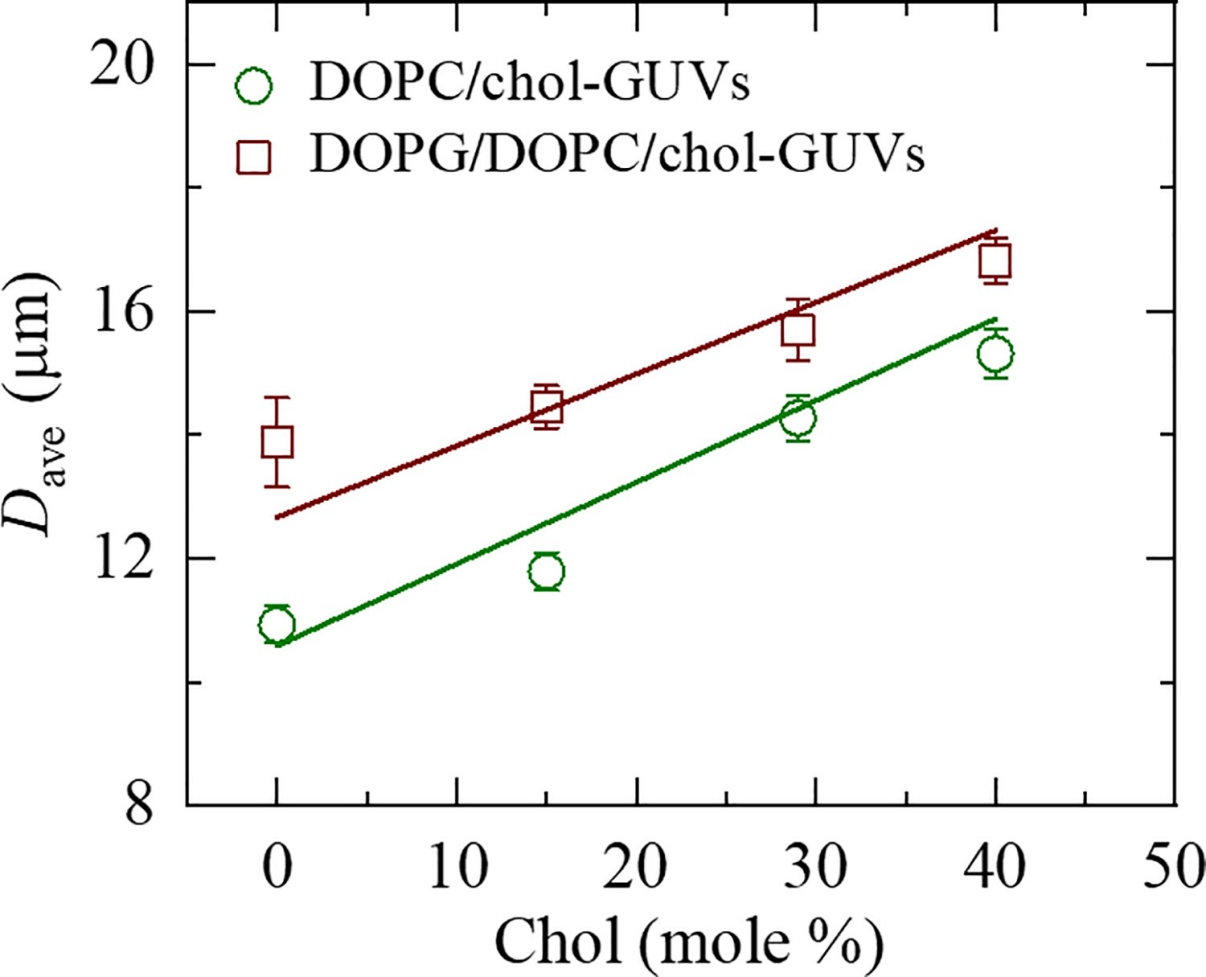
The cholesterol concentration dependent average size of DOPC/chol-GUVs and DOPG/DOPC/chol-GUVs. Average values and standard errors of the size for each membrane were obtained from 12–16 independent experiments using 350 GUVs in each experiment. The solid lines show the theoretical curve corresponding to the Eqs 12, 13 and 14. The fitting parameters for DOPC/chol-GUVs are *D*_freqPC_ = 15.8 μm, *K*_DOPC_ = 18.0 *k*_B_*T* and *η* = 0.45 *k*_B_*T* and for DOPG/DOPC/chol-GUVs are *D*_freqPC_ = 18.9 μm, *K*_DOPC_ = 18.0 *k*_B_*T*, *η* = 0.33 *k*_B_*T* and *γ* = 2.9 *k*_B_*T*/mM^3/2^. The values of *R*^2^ are obtained 0.99 and 0.62 for DOPC/chol-GUVs and DOPG/DOPC/chol-GUVs, respectively.

The dependence of *D*_ave_ on *C*_h_ for cholesterol-rich neutral GUVs and cholesterol-rich charged GUVs is shown in Fig 4. It is seen that as the cholesterol concentration in GUVs membrane increases, the average size of the GUVs increases for both the neutral and charged membranes. So, our results show that the sizes of self-assembled neutral and charged GUVs depend on the cholesterol concentration in vesicle membranes. Generalizing these results, one can conclude that with the increase of cholesterol, the fraction of large GUVs in the population of vesicles increases. We will discuss these results in framework of the theory which describes the behavior of the system as interplay between the entropy of the system and bending energy of lipid membrane. It is worth to note that our results correspond to experimental investigations of the effects of cholesterol on the size of sonicated phospholipid vesicles where 10% cholesterol caused a 30% increase in the surface area of vesicles [66].

The bending moduli of membranes for different cholesterol containing neutral and charged GUVs were estimated from the theoretically fitted histograms (using Eq 7) of Figs 2 and 3. The obtained values of *K*_ben_ for different conditions are shown in Table 1 where *K*_ben_ increases with the increase of cholesterol. Some literature values of bending modulus are also presented in Table 1 for comparison. Our results are well supported by the recent report [44].

**Table 1:** A summary of the data for bending modulus of cholesterol containing membranes.

Membrane composition	Temperature	Measuring technique	Bending modulus	Ref.
			*K*_ben_ (*k*_B_*T*)	
DOPC/chol	25°C	Neutron spin echo (NSE) spectroscopy		[44]
(50 nm vesicles)				
100/0			13.01 ± 0.37	
80/20			18.09 ± 0.64	
70/30			23.15 ± 0.58	
60/40			30.31 ± 1.04	
50/50			38.81 ± 1.63	
DOPC/chol				
(100 nm vesicles)				
100/0			19.05 ± 0.65	
90/10			22.46 ± 1.77	
80/20			30.34 ± 2.47	
DOPC/chol (bilayers)	25°C	Real-space fluctuation (RSF) -MD Simulation		
100/0			18.3 ± 0.3	
90/10			22.5 ± 0.4	
80/20			30.5 ± 0.6	
70/30			38.0 ± 0.6	
60/40			52.1 ± 0.8	
50/50			67.7 ± 1.1	
DOPC/chol (bilayers)	25°C	Buckling Simulations		[76]
100/0			21.2 ± 1.0	
80/20			26.8 ± 1.1	
60/40			31.6 ± 0.9	
DOPC/chol-GUVs	25°C	Size distribution analysis		This work
100/0			18.0 ± 0.9	
85/15			23.5 ± 0.3	
71/29			28.1 ± 0.3	
60/40			31.2 ± 0.3	
DOPG/DOPC/chol-GUVs				
70/30/0			25.9 ± 0.5	
46/39/15			26.9 ± 0.3	
43/28/29			32.1 ± 0.4	
40/20/40			37.5 ± 0.6	
POPC/chol-GUVs	25°C	Size distribution analysis		This work
100/0			18.5 ± 0.6	
85/15			23.3 ± 0.8	
71/29			28.2 ± 0.6	
60/40			31.1 ± 0.5	
POPC/chol	22°C	Neutron spin echo and dynamic light scattering		[82]
(100 nm vesicles)				
100/0			19.0 ± 2.0	
90/10			20.0 ± 2.0	
80/20			23.0 ± 2.0	
60/40			27.0 ± 2.0	
50/50			37.0 ± 2.0	
DOPC/chol-GUVs	15°C	Micropipette aspiration		[8, 85]
100/0			20.7 ± 2.4	
DOPC/chol-GUVs				
100/0			21.9 ± 1.4	
50/50			80.2 ± 5.8	
DOPC/chol-GUVs	22°C	Micropipette aspiration		[49]
67/33			20.9 ± 3.2	
50/50			22.1 ± 8.1	
DOPC/chol	30–33°C	X-ray diffuse scattering		[41, 86]
(stacks of bilayers)				
100/0			18.25	
90/10			16.79	
80/20			17.52	
70/30			18.01	
60/40			17.76	
50/50			23.36	
DOPC/chol-GUVs	22°C	Micropipette aspiration		[48]
100/0			16 ± 2	
67/33			15 ± 2	
DOPC/chol-GUVs	23°C	Shape fluctuation spectroscopy		[42]
100/0			26.3 ± 2.4	
90/10			28.2 ± 3.2	
80/20			27.5 ± 2.9	
70/30			22.4 ± 3.2	
56/44			22.9 ± 1.7	
50/50			26.3 ± 1.9	
DOPC/chol-GUVs	23°C	Electrodeformation		[42]
100/0			~ 23.4	
90/10			~ 21.4	
80/20			~ 26.8	
70/30			~ 25.8	
DOPC/chol-GUVs	25°C	Fluctuation spectroscopy		[87]
100/0			~ 22.0	

## 4 Theory

Recently, we have developed a theory for the formation of GUVs in population [25]. The theory was developed by considering the dynamics of vesicle formation from lipid bilayer aggregates. At first, we will give a very brief description of this theory. Then, the theory is extended for neutral and charged membranes containing various concentrations of cholesterol. We postulate the existence of some initial population of lipid bilayer aggregates which is treated as population of *N*_init_ lipid supramolecular structures. All such aggregates in this initial population are assumed to be exactly the same. The surface area of each aggregate is *S*_init_. The initial population transforms into population of different sizes GUVs in which the final equilibrium distribution of GUVs by the sizes achieved. Each GUV in this population is described by a number of initial aggregates *m*, which composes it. Because the total surface of *m* initial aggregates is *mS*_init_, the diameter of *m*-GUV (i.e., composed of *m* initial aggregates) is $D_{m}=\sqrt{mS_{\mathrm{inst}}/\pi }$. The state of such system is determined by the Helmholtz free energy as follows [24, 25]:

$$\frac{G(n_{m},m)}{k_{B}T}=(4\pi K_{\mathrm{ben}}\sum_{m=1}^{N_{\mathrm{init}}}n_{m})-[N_{\mathrm{init}}\ln\left(\phi N_{\mathrm{init}}\right)-\sum_{m=1}^{N_{i\mathrm{nit}}}n_{m}\ln(mn_{m})]$$ (3)

here *n*_m_ is the number of *m-*GUVs in the system, *K*_ben_ is the bending modulus of membranes in *k*_B_*T* unit where *k*_B_ is the Boltzmann constant and *T* is the absolute temperature, and *ϕ* is the volume fraction of initial vesicles in solution. The first term in Eq 3 describes the bending energy [67] of all vesicles in the GUVs population and the second term is the contribution of configurational entropy. In the general case, the bending energy of the lipid bilayer consists of a number of components [30, 67–71]. In the case of giant unilamellar lipid vesicles considered here, all these components can be described by a single generalized parameter *K*_ben_. In our case, there are no problems associated with spontaneous curvature since we consider only unilamellar vesicles (with a symmetric bilayer) for which the total spontaneous curvature is zero. As for the component associated with the Gaussian one, in our case this component is summed up with the component describing the local curvature, since both the radii of GUV’s principal curvatures coincide. Eq 3 determines the free energy of the GUVs population for any arbitrary set of {*n*_m_, *m*}. The equilibrium state of the population is determined by equation

$$\frac{\partial G(n_{m},m)}{\partial n_{m}}=0\;\mathrm{for}\;(n_{m}=n_{m1},n_{m2},n_{m3},\ldots \ldots )$$ (4)

$$\mathrm{under}\;\mathrm{condition}\;\sum_{m=1}^{N_{\mathrm{init}}}n_{m}m=N_{\mathrm{init}}$$ (5)

The solution of Eqs 4 and 5 is as follows,

$$n_{m}\left(D_{m}\right)=N_{\mathrm{init}}\phi \frac{S_{\mathrm{init}}}{\pi D_{m}^{2}}\exp[-\frac{4S_{\mathrm{init}}}{D_{m}^{2}}K_{\mathrm{ben}}]$$ (6)

The non-measurable parameter *m* in Eq 6 is converted to the measurable one, namely to the size of *m*-GUVs, *D*_m_, by using expressions $D_{m}=\sqrt{mS_{\mathrm{init}}/\pi }$ and $D_{\mathrm{freq}}=2\sqrt{S_{\mathrm{init}}K_{\mathrm{ben}}}$. The latter expression is obtained from the condition $\frac{\partial n_{m}}{\partial D_{m}}=0$. The parameter *D*_freq_ is the mode of the distribution or in other words, the most frequent diameter that can be obtained from experimental histograms. Then one can obtain from Eq 6 the probability density function *f(D*_m_*)* (i.e. histogram) as follows,

$$f\left(D_{m}\right)=\frac{n_{m}(D_{m})}{\Delta D_{m}}=(\frac{L}{K_{\mathrm{ben}}}){\left(\frac{D_{\mathrm{freq}}}{D_{m}}\right)}^{2}\exp[-{\left(\frac{D_{\mathrm{freq}}}{D_{m}}\right)}^{2}],$$ (7)

where the step of experimental histogram Δ*D*_m_ = 2 μm and *L* = *N*_init_*ϕ*/(4*π*Δ*D*_m_). Eq 7 has two fitting parameters, *D*_freq_, and *K*_ben_ (*L* is normalized parameter). The value of *K*_ben_ can be determined in each specific experiment from the fitting of the corresponding histogram (see also the legends in Figs 2 and 3). Using the Eq 7, one can also determine the average size of GUVs in population as follows [72]:

$$D_{\mathrm{ave}}=\int_{0}^{\infty }D_{m}f(D_{m})dD_{m}=-(\frac{LD_{\mathrm{freq}}^{2}}{2K_{\mathrm{ben}}})Ei[-{\left(\frac{D_{\mathrm{freq}}}{D_{\max}}\right)}^{2}]$$ (8)

here *Ei*(*z*) is the exponential integral function, *D*_max_ is the size of the greatest vesicle obtained in experiment. This equation is not convenient for an analysis of the GUV’s parameters influencing the *D*_ave_. Hence it is worth to present this equation in a form that will make it relatively easy to analyze the influence of the system parameters on the distribution of vesicles by size. Because Eq 7 gives the positively skewed distributions, we approximate the Eq 7 by lognormal distribution (see Eq 1), which also gives the positively skewed distributions. By comparing Eqs 1 and 7 and using certain conditions, we obtain after manipulation as follows [25].

$$D_{\mathrm{ave}}=\exp(\mu +\frac{1}{2}\sigma^{2})=D_{\mathrm{freq}}\exp(\frac{3}{2}\sigma^{2})=D_{\mathrm{freq}}b,$$ (9)

where *D*_freq_ = exp(*μ*−*σ*^2^) and *b* = exp(3*σ*^2^/2) = 0.67.

Now we extend the previously developed theory [25] for the cholesterol containing membranes and discuss the main findings in sections 5.1 and 5.2 based on the theory. As it was discussed above the key physical parameter influencing the GUVs size distribution (and therefore, on the average size *D*_ave_) is *K*_ben_. We obtained *K*_ben_ in our experiments for *C*_h_ = 0 from the fitting of experimental results by theoretical Eq 7 in the range of 18.0−37.4 *k*_B_*T* (see Table 1). These values are at the same order as the values of *K*_ben_ for PC membrane [8] and for PG/PC membrane [25, 67, 73]. Therefore, the value of *D*_ave_ is determined by *D*_freq_ which, in turn, is determined by *K*_ben_ (see above $D_{\mathrm{freq}}=2\sqrt{S_{\mathrm{init}}K_{\mathrm{ben}}}$). We postulate for simplicity that *S*_init_ does not depend on cholesterol concentration, while at the same time *K*_ben_ depends on *C*_h_. It means that in our model *D*_ave_ is proportional only to $\sqrt{K_{\mathrm{ben}}}$ i.e.


$$D_{\mathrm{ave}}=(2b\sqrt{S_{\mathrm{init}}})\sqrt{K_{\mathrm{ben}}}=\mathrm{const}\sqrt{K_{\mathrm{ben}}}$$ (10)

**Fig 5:**
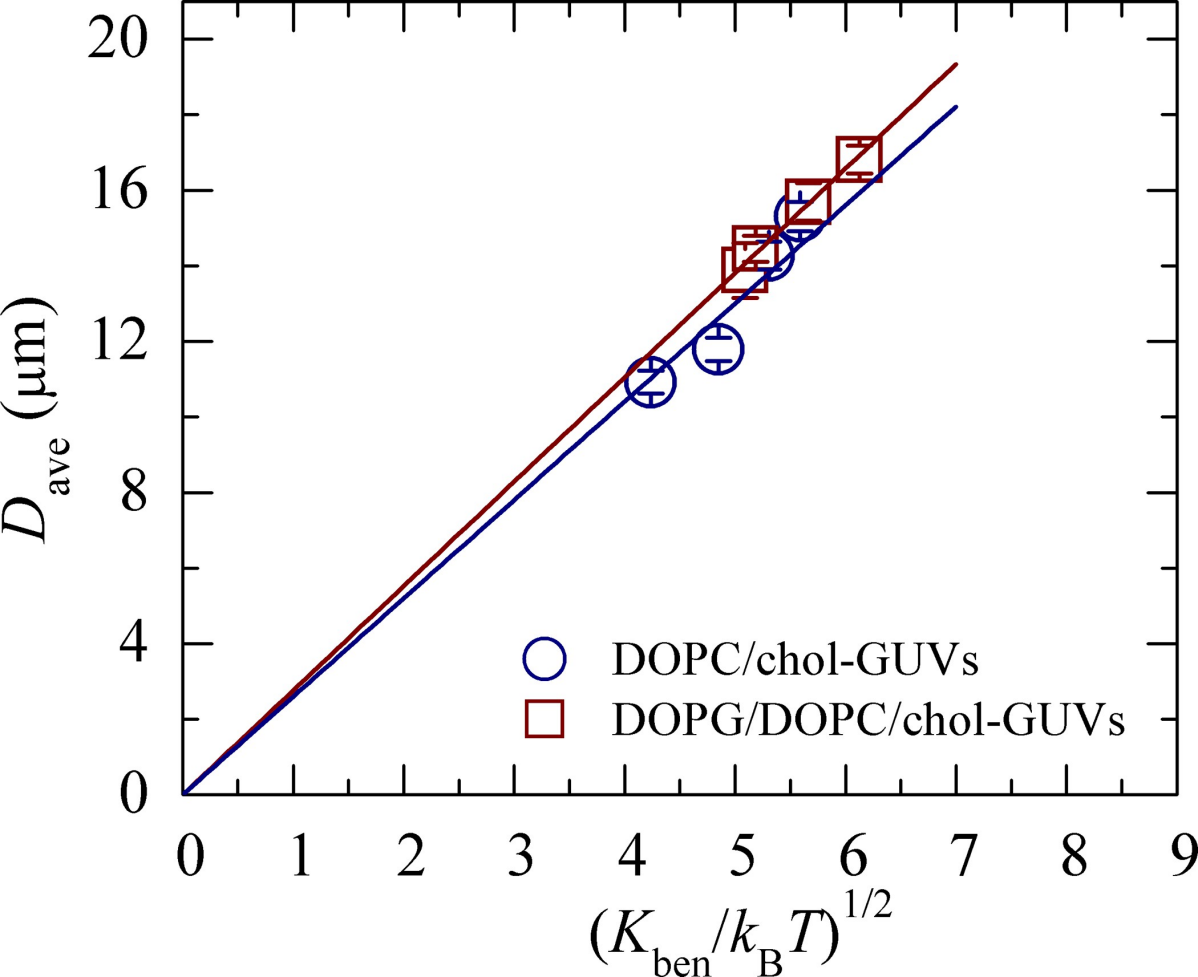
Relationship between the average size and the $\sqrt{K_{\mathrm{ben}}}$ of cholesterol containing neutral and charged membranes. Average values and standard errors are determined from 12–16 independent experiments.

This theoretical result is supported by our experiments (see Fig 5).

We have shown earlier that electrical charge of the vesicle membrane increases the bending modules of membrane *K*_ben_ [25]. The results of the current research show that the cholesterol also increases *K*_ben_. Hence one can consider as a first approximation that the bending modulus of cholesterol-rich membranes can be written as sum of the three components, namely as follows,

$$K_{\mathrm{ben}}=K_{\mathrm{DOPC}}+K_{\mathrm{ben}}^{\mathrm{el}}+K_{\mathrm{ben}}^{\mathrm{ch}},$$ (11)

where *K*_DOPC_ is the bending modulus of pure DOPC membrane (i.e., without cholesterol), $K_{\mathrm{ben}}^{\mathrm{el}}$ indicates the surface charge density term of bending modulus and $K_{\mathrm{ben}}^{\mathrm{ch}}$ indicates the cholesterol term of the modulus. Inserting Eq 11 in Eq 10 one obtains the expression for *D*_ave_ as follows,

$$\begin{array}{l} D_{\mathrm{ave}}=bD_{\mathrm{freq}}=2b\sqrt{(K_{\mathrm{DOPC}}+K_{\mathrm{ben}}^{\mathrm{el}}+K_{\mathrm{ben}}^{\mathrm{ch}})S_{\mathrm{init}}}\\ =2b\sqrt{K_{\mathrm{DOPC}}S_{\mathrm{init}}}\sqrt{1+\frac{K_{\mathrm{ben}}^{\mathrm{el}}+K_{\mathrm{ben}}^{\mathrm{ch}}}{K_{\mathrm{DOPC}}}}\\ =bD_{\mathrm{freqPC}}\sqrt{1+\frac{K_{\mathrm{ben}}^{\mathrm{el}}+K_{\mathrm{ben}}^{\mathrm{ch}}}{K_{\mathrm{DOPC}}}}\approx bD_{\mathrm{freqPC}}\left(1+\frac{K_{\mathrm{ben}}^{\mathrm{el}}+K_{\mathrm{ben}}^{\mathrm{ch}}}{2K_{\mathrm{DOPC}}}\right), \end{array}$$ (12)

where $D_{\mathrm{freqPC}}=2\sqrt{K_{\mathrm{DOPC}}S_{\mathrm{init}}}$ is the most frequent vesicle size in case of DOPC-GUVs. It was considering in Eq 12 that $(K_{\mathrm{ben}}^{\mathrm{el}}+K_{\mathrm{ben}}^{\mathrm{ch}})/K_{\mathrm{DOPC}}<1$ in the total range of the cholesterol concentrations used in our experiments.

## 5 Discussion

### 5.1 Average size of GUVs and the bending modulus of membranes

As it follows from Eq 10 the average size of GUVs in the population is determined by the value of *K*_ben_. Fig 5 shows the dependence of *D*_ave_ on $\sqrt{K_{\mathrm{ben}}}$, drawn on the basis of Table 1. It is seen from this figure that *D*_ave_ can be approximated by a linear function of $\sqrt{K_{\mathrm{ben}}}$. The appropriate constants obtained from Fig 5 are 2.60 [μm/(*k*_B_*T*)^1/2^] for cholesterol-rich neutral membranes and 2.76 [μm/(*k*_B_*T*)^1/2^] for cholesterol-rich charged membranes. Hence, one can conclude that the above-mentioned assumption (see Eq 10) is valid. Moreover, increase of *K*_ben_ results the increase of the average size of GUVs in population. The explanation of such behavior of the system under consideration is the following. As it is seen from Eq 3 increase of *K*_ben_ means the increase of the total elastic energy of the population. To decrease the free energy in equilibrium, state the system “trends” to increase the number of large vesicles, because the elastic energy of such vesicles is less than the same of small ones. Hence, the size distribution of GUVs is determined by *K*_ben_. The experimental data for the bending rigidity of DOPC/chol and DOPG/DOPC/chol membranes is presented in Table 1. The temperature and various measuring techniques are included in the columns.

The problem of the influence of cholesterol on the bending modulus of DOPC membrane has attracted the attention of a number of research groups in the last decade [74]. In our experiment, the bending rigidity increased with the increase of cholesterol content in the DOPC membranes. However, in experiments based on tube pulling [48, 49], shape analysis and electrodeformation of giant vesicles [42], and X-ray diffuse scattering fluctuations in bilayer stacks [41] reported that the bending modulus of DOPC bilayers, unlike other standard lipids, does not increase with addition of cholesterol. From another side, a recent paper [44] based on neutron spin echo (NSE), NMR relaxation spectroscopy and RSF-MD simulations claims that the bending modulus of DOPC membrane increases three-fold for cholesterol mole fractions of 50%. NSE spectroscopy has been frequently used to determine the bending rigidity of lipid bilayer. Zilman and Granek [75] first introduced a numerical prefactor 0.025 in the theory that connects the bending rigidity. Then, prefactor 0.0058 was calculated by assuming the ratio (0.6) between the distance of the neutral surface from the bilayer midplane and the thickness of the bilayer. But, this moves the neutral surface into the headgroup region of the bilayer. Recently, Gupta et al [74] compiled many results and converted all the bending moduli to the most recent prefactor 0.0069 by considering the ratio 1.0 instead of 0.6. In the paper [44], the prefactor was used 0.0069 which we added in the Table 1. For increase of cholesterol content from 0 to 40% mole fraction in the membranes of vesicles, the bending modulus of DOPC bilayers increases from 21.2 ± 1.0 to 31.6 ± 0.9 *k*_B_*T* obtained using buckling simulations [76]. One can see from the Table 1 that our results are very consistent with these results [44, 76].

In the electrodeformation [42], the factors influencing the measurement of bending rigidity are i) the shielding of applied electric field to GUVs due to the presence of many different sized vesicles in the solution, ii) the inhomogeneous stress distribution in the membranes of GUVs due to the chamber geometry, iii) the location of selected GUVs with respect to the two cylindrical electrodes, and iv) the dielectrophoretic motion. GUVs may exhibit such motion due to the application of nonuniform electric field. In the shape fluctuation (flicker spectroscopy), the factors affecting to determine the bending rigidity are i) the resolution of camera as used in the experiment, and ii) the low contrast between the vesicles and its surrounding. These possible sources may differentiate the previous results [42] from our investigations.

It is worth to discuss shortly the terms of Eq 12. The parameter *b* was obtained above (see Eq 9), $D_{\mathrm{freqPC}}=2\sqrt{K_{\mathrm{DOPC}}S_{\mathrm{init}}}$ can be obtained from experimental histograms. As for $K_{\mathrm{ben}}^{\mathrm{el}}$ we have discussed in details in our previous paper [25] and we have concluded that this term can be determined as following,

$$K_{\mathrm{ben}}^{\mathrm{el}}=\mathrm{const}\frac{\Omega_{\mathrm{ch}}^{2}}{\kappa^{3}}=\frac{\gamma X^{2}}{\sqrt{C^{3}}{\left(1-c_{h}+\beta c_{h}\right)}^{2}}=\frac{\gamma X^{2}}{\sqrt{C^{3}}{\left(1-0.5c_{h}\right)}^{2}},$$ (13)

where *β* = *a*_ch_/*a*_DOPG_ = 0.5, Debye length $\kappa^{-1}=\sqrt{\frac{k_{B}T\varepsilon_{s}\varepsilon_{0}}{e^{2}C}}=\frac{A}{\sqrt{C}}=0.76\;[\mathrm{nm}]$ where *C* = 162 mM is the bulk concentration of NaCl in buffer, *ε*_S_ is the dielectric constant of solution, *ε*_0_ is the vacuum permittivity, *k*_B_ is the Boltzmann constant and *T* is absolute temperature. *A* = 0.304 [nm×mM^1/2^] and *κ* ^-1^ = 0.76 [nm] for *C* = 162 mM used in our experiment.

We use this equation for interpretation and fitting of our results (Fig 4) in which *γ* = 2.9 *k*_B_*T*/mM^3/2^ used as a fitting parameter for *X* = 0.70. To get the information about $K_{\mathrm{ben}}^{\mathrm{ch}}$, it is necessary to analyze the experimental results for cholesterol-rich neutral GUVs. Let us describe the dependence of *K*_ben_ on *C*_h_ as following *K*_ben_ = *K*_DOPC_+*ηC*_h_. Based on experiment results one obtains *K*_DOPC_ = 18.0 *k*_B_*T* and *η* = 0.45 *k*_B_*T*. Hence it can be written as follows,

$$K_{\mathrm{ben}}^{\mathrm{ch}}=\eta C_{h}$$ (14)

### 5.2 Size distribution histograms fitting

It is seen from the histograms of Figs 2 and 3 that with the increase of cholesterol the histogram peak shifts in the range of larger vesicles, indicating the decrease of histogram asymmetricity. In Figs 2B, 2D and 3B, 3D, the red lines show the theoretical distribution obtained using Eq 7. It can be seen that the theoretical curves describe the experimental histograms well. However, as it is seen from a comparison of the theoretical equation with the experimental results, the theoretical distribution overestimates slightly the number of vesicles in the region of vesicle large sizes. This is due to the fact that the Eq 7 takes into account only configurational entropy, but not the orientational one. Since this equation is enough to describe the specific distribution as well as the histogram transformations upon change of cholesterol and to avoid cumbersome mathematical expressions, we do not consider here the orientational entropy. It is to be noted that the energetics generally contain both the bending modulus and Gaussian modulus (i.e., 2*K*_ben_+*K*_Gauss_). Since Gaussian energy makes no difference for shape changes in a single vesicle, as for simplicity we ignored it for the determination of *k*_ben_ where it was assumed that *K*_ben_/*K*_Gauss_ = -1, which is similar to that used before [24].

The average value of *K*_ben_ are obtained (18.0 ± 0.9) *k*_B_*T* for DOPC/chol (100/0)-GUVs (i.e. DOPC-GUVs without cholesterol), which is very close to the value (20 ± 0.5) *k*_B_*T* for DOPC-GUVs obtained in micropipette aspiration technique [8]. In addition, *K*_ben_ for DOPG/DOPC(70/30)-GUVs is close to the value obtained in our previous study [25]. The average values of *K*_ben_ at various cholesterol concentrations are provided in Table 1. Our results demonstrate that as the cholesterol increases in the DOPC or DOPG/DOPC-GUVs, the value of *K*_ben_ increases.

Taking into account of that from one hand we have shown that the size distribution and average size of GUVs are determined by *K*_ben_ (Eqs 7 and 10), and from the other, our experimental results demonstrated significantly dependences of GUVs size on cholesterol content (see Figs 2 and 3). Therefore, we can say that our results also exhibit the dependence of the vesicle membrane bending modulus on cholesterol.

As we have discussed above the histograms of Figs 2 and 3 show that with the increase of cholesterol concentration in vesicles membranes the histogram peak shifts in the range of larger vesicles. This means that the number of large vesicles in the system increases in GUVs population with the addition of cholesterol. As a result, the value of *D*_ave_ also increases. The physical explanation of such behavior is simple. As we discussed above, the cholesterol induces increase of bending modulus of membranes. Hence the energy term of free energy increases (see Eq 3). However, it is seen that this term does not contain the vesicle size explicitly. Then, a question can arise how this term can describe the influence of bending modulus influence on vesicle size distribution. To understand this, it is necessary to take into account that this term contains *n*_m_ vesicles composed with *m* initial aggregates. The total number of vesicles in the system $\sum_{m=1}^{N_{\mathrm{init}}}n_{m}=N_{\mathrm{ves}}$ is not fixed. The greater fraction of large vesicles is in the population, the smaller is *N*_ves_. Hence the system tends to decrease the energy term in Eq 3 by decreasing the total number of vesicles *N*_ves_. Smaller number of vesicles means the greater fraction of large vesicles and consequently larger average size of vesicles in the population.

The solid lines in Fig 4 demonstrate the theoretical curves corresponding to Eqs 12, 13 and 14 for the dependence of *D*_ave_ on cholesterol for neutral and charged membranes. It is seen the satisfactory fitting of theoretical curves (solid line) to the experimental data. This means that the theory describes the real processes satisfactory in the system under consideration.

In our investigations, we could measure the GUVs with diameters greater than 3 μm without any difficulties. Hence, we omitted to count the vesicles with diameters less than 3 μm. It is to be noted that the range of the diameters of GUVs were 3.3–40.9 μm and 6.3–50.2 μm as shown in Figs 2A, 2B and 2C, 2D, respectively. On the other hand, the ranges of the diameters of GUVs were 5.6 − 50.9 μm and 5.5–72.3 μm as shown in Figs 3A, 3B and 3C, 3D, respectively. The same technique was used in our recent papers to measure the similar range of size distribution of GUVs [7, 25, 64, 65]. In addition, another group was able to measure the GUVs with diameters greater than 2 μm using the similar technique [77].

In these investigations, we prepared two types of membranes. One is cholesterol containing neutral membranes such as DOPC/chol-GUVs and another is cholesterol containing charged membranes such as DOPG/DOPC/chol-GUVs. The DOPG/DOPC/chol-GUVs were prepared in PIPIES buffer. The internal solution of the DOPG/DOPC/chol-GUVs was 0.10 M sucrose containing PIPES buffer and the external solution of the same GUVs was 0.10 M glucose containing PIPES buffer. As the preparation of neutral GUVs in PIPES buffer is difficult, we used the MilliQ water instead of PIPES buffer. The internal solution of the DOPC/chol-GUVs was 0.10 M sucrose containing MilliQ water and the external solution of the same GUVs was 0.10 M glucose containing MilliQ water. So, we used 0.10 M glucose in both neutral and charged membranes. We compared the bending modulus of cholesterol containing various neutral GUVs. We also compared the bending modulus of cholesterol containing various charged GUVs. If the 0.10 M glucose is used with PIPES buffer instead of MilliQ water, the bending modulus may change. However, that is not the main focus of this research. Our aim is to investigate the change in bending modulus by changing the cholesterol content in the neutral and charged GUVs. Recently, we investigated the change of bending modulus by changing the salt concentration in PIPES buffer solution and obtained that as the salt concentration in buffer increases the bending modulus of membranes decreases [25].

### 5.3 Estimation of area compressibility modulus of cholesterol-rich neutral membranes

One more interesting characteristic of lipid membranes that has to be discussed here is the area compressibility modulus *K*_A_ which is connected with *K*_ben_ [8, 78, 79]. The polymer brush theory defines this relationship as follows:

$$K_{A}=\frac{24K_{\mathrm{ben}}}{{\left(h-h_{e}\right)}^{2}}$$ (15)

where *h* is the bilayer thickness (~ 4 nm), *h*_e_ is the head group thickness (~ 1 nm). The influence of cholesterol on area compressibility modulus was considered in different studies. Particularly, Pan et al obtained the increasing trend in area compressibility modulus due to the incorporation of cholesterol in DOPC membranes and the values of *K*_*A*_ for DOPC/chol (100/0), DOPC/chol (70/30) and DOPC/chol (50/50)-GUVs were obtained 290, 420 and 840 mN/m, respectively [41]. Incorporation of cholesterol in SOPC-GUVs (which is very similar to DOPC-GUVs) increased the area compressibility modulus greatly [80]. The value of area compressibility modulus was reported (193 ± 20) mN/m for SOPC-GUVs. Besides, upon addition of 14, 28, 38 and 43% cholesterol in SOPC-GUVs the area compressibility modulus increased to (216 ± 12), (244 ± 24), (333 ± 9) and (609 ± 44) mN/m, respectively. Summarizing these results one can conclude that as the cholesterol increases the values of *K*_A_ increase. Our results dealing with the estimation of *K*_ben_ (see Table 1) give the opportunity to get the *K*_A_ in accordance with the Eq 15. We have done proper estimations and obtained the following. The values of *K*_A_ for DOPC/chol (100/0), DOPC/chol (85/15) and DOPC/chol (71/29) and DOPC/chol (60/40)-GUVs are obtained (197 ± 10), (258 ± 4), (308 ± 4) and (342 ± 3) mN/m, respectively. Therefore, the value of *K*_A_ for DOPC/chol (100/0) is very similar to that found in micropipette aspiration technique (230 ± 10) mN/m [8]. Hence, our estimations on area compressibility modulus for cholesterol-rich neutral membrane based on our experimental measurements are very similar to the reported ones.

### 5.4 Bending modulus of POPC/chol membranes

To confirm the validity of this study, it is very important to investigate the size distribution of POPC/chol-GUVs and then estimate the bending modulus of membranes. The value of bending modulus of POPC-GUVs was obtained 18.5 ± 0.6 *k*_B_*T*, which showed good agreement with the literature value [81]. This estimated value corresponds to a fluid lipid bilayer that is relatively flexible upon bending. In contrast, investigations at the higher cholesterol content in the membranes of vesicles indicate a substantial stiffening; for example, the bending modulus was obtained 31.1 ± 0.5 *k*_B_*T* at 40 mol% cholesterol. These results well support to the literature values in which cholesterol content in POPC lipids stiffens the membranes [82, 83]. The increase of bending modulus due to the increase of cholesterol content is compatible with the well-known structural condensation on the disordered phases of fluid phospholipids [84]. As the increasing trend of bending modulus due to cholesterol content in POPC vesicles is well established and without controversy [82, 83]. Therefore, our study on POPC/chol gives us the confidence for estimating the bending modulus of DOPC/chol membranes. The sizes of GUVs were obtained 12.0 ± 1.2, 13.9 ± 1.1, 14.9 ± 1.1 and 16.2 ± 2.5 μm for 0, 15, 29 and 40 mole% cholesterol in POPC vesicles, respectively. The values of bending rigidity of DOPC/chol, DOPG/DOPC/chol and POPC/chol membranes are presented in Table 1.

Appropriate amounts of cholesterol in cell membranes are essential for regulating various biological functions such as how viruses spread and how cells divide. The measurement of bending modulus of membranes prepared by a mixture of a common type of lipid DOPC and cholesterol and also a mixture of DOPG, DOPC and cholesterol provides the information on how membranes bend during different functions. These investigations show that increase of cholesterol in both neutral and charged membranes increases the bending rigidity. The tighter packing of molecular building blocks results in stiffer membranes that cannot bend so easily. Cholesterol stiffens DOPC membranes on the local scales accessible by the techniques reported earlier [44]. These results supported the universal stiffening effects of cholesterol on lipid membranes. As DOPC and DOPG/DOPC are the synthetic representative of the class of phospholipids, these findings are highly important for understanding the ideal circumstances. The semi-rigid structure of cell membranes is highly preferable for maintaining a suitable structure and for showing the flexible nature to permit dynamic movement of signaling proteins along with functional domains. The results would be helpful to design the drug for the treatment of diseases and various biological anomalies. As for example, how cholesterol affects the budding of membrane in the maturation of viruses (i.e., HIV, coronavirus) is a crucial question with earnest socio-economic and scientific impact.

## 6 Conclusions

We investigated the influence of different fractions of cholesterol in the neutral and charged GUV’s membranes on the size distribution of vesicles in the population. A method for determining the bending modulus of lipid membranes based on the analysis of such distributions has been proposed. The GUVs size distributions were presented as a set of histograms. The obtained histograms are well described by the classical lognormal distribution with positively skewed asymmetry. Such distribution manifests that the number of GUVs with sizes smaller than the average one prevails over the number of vesicles with a size larger than the average size. We have obtained that with the increase of cholesterol fraction in vesicles membranes, the peak of the histograms shifts to right, i.e., in the region of large vesicles for both the neutral and charged GUVs. Hence, with the increasing of cholesterol content, the fraction of large GUVs in the population increases. The theory, developed by the framework of Helmholtz free energy of the system, from one hand describes the experimental results satisfactorily and from another hand gives the opportunity to estimate the influence of cholesterol on the bending modulus of lipid membranes. In our investigation, the specific size distribution is determined by the bending modulus of membranes of GUVs. Moreover, bending modulus increases with the increase of cholesterol content in the neutral membranes as well as of charged membranes. The proposed method for the estimation of bending modules gives the opportunity to clarify the influence of cholesterol on the mechanical characteristics of lipid vesicles.
